# Liposomal Encapsulation of Pine Green Cone Essential Oil: The Influence of the Carrier on the Enhancement of Anti-Inflammatory Activity

**DOI:** 10.3390/pharmaceutics17091182

**Published:** 2025-09-11

**Authors:** Snježana Mirković, Vanja Tadić, Marina Tomović, Anica Petrović, Marijana Andjić, Jovana Bradić, Sanja Perać, Aleksandar Radojković, Jelena Jovanović, Ana Žugić

**Affiliations:** 1 Public Health Institution Hospital “Sveti Vračevi”, Srpske Vojske 53, 76300 Bijeljina, Bosnia and Herzegovina; snjezanam1983@gmail.com; 2 Department of Pharmaceutical Research and Development, Institute for Medicinal Plants Research “Dr. Josif Pančić”, 1 Tadeuša Košćuška St., 11000 Belgrade, Serbia; vtadic@mocbilja.rs; 3 Department of Pharmacy, Faculty of Medical Sciences, University of Kragujevac, 69 Svetozara Markovica St., 34000 Kragujevac, Serbia; marinapop@gmail.com (M.T.); andjicmarijana10@gmail.com (M.A.); jovanabradickg@gmail.com (J.B.); 4 Center for Molecular Medicine and Stem Cell Research, Faculty of Medical Sciences, University of Kragujevac, 69 Svetozara Markovica, 34000 Kragujevac, Serbia; 5 Center of Excellence for Redox Balance Research in Cardiovascular and Metabolic Disorders, 69 Svetozara Markovica St., 34000 Kragujevac, Serbia; 6 Institute for Multidisciplinary Research, University of Belgrade, 1 Kneza Višeslava St., 11030 Belgrade, Serbia; sanjaprsic@gmail.com (S.P.); aleksandarrr@yahoo.com (A.R.); jelenajovanovic208@gmail.com (J.J.)

**Keywords:** essential oil chemical profile, liposomes, carrageenan-induced rat paw edema model, physico-chemical properties

## Abstract

**Background/Objectives:** This study aimed to investigate the traditionally claimed anti-inflammatory effect of essential oil (EO) derived from pine green cones per se and after encapsulation into liposomes, which is expected to enhance its bioactivity and stability. **Methods**: The chemical profiling of EO was conducted using GC/GC-MS. The physico-chemical characterization of the liposomal formulation (LEO) included encapsulation efficiency, FTIR spectroscopy, and AFM imaging. Additionally, parameters such as mean particle diameter, polydispersity index, zeta potential, pH, and electrical conductivity were evaluated and reassessed after 30 days and 1 year to determine formulation stability. The in vivo anti-inflammatory effect of the EO and LEO was examined using a carrageenan-induced rat paw edema model. **Results**: The *Pinus halepensis* EO contained 14 components, mainly, α-pinene, myrcene, and (E)-caryophyllene. Encapsulation efficiency was 97.35%. AFM analyses confirmed the nanoscale dimensions and spherical shape of liposomes, while FTIR indicated successful encapsulation through overlapping functional groups. The droplet size of blank liposomes (L) ranged from 197.4 to 217 nm, while adding the EO decreased the droplet size and electrical conductivity. The polydispersity index (PDI) remained below 0.2. The zeta potential of the liposomes was between −35.61 and −49.43 mV, while the pH value was in the range of 4.35 to 5.01. These results indicate satisfactory stability across repeated measurements. Administration of LEO significantly inhibited paw edema relative to the controls, with a percentage inhibition of approximately 69%, which does not significantly differ from the effect of hydrocortisone, which was used as a positive control. **Conclusions**: This is the first study to report liposomal encapsulation and in vivo anti-inflammatory activity of an EO derived specifically from green cones of *P. halepensis*. Our findings demonstrate that EO-loaded liposomes exhibited favorable physico-chemical properties and notable anti-inflammatory activity, comparable to that of hydrocortisone. These results support their potential application in the development of effective topical anti-inflammatory formulations.

## 1. Introduction

Pines (species from the *Pinus* genera, Pinaceae) have a long history of ethnotherapeutic application in wound healing and treatment of various skin conditions (eczema, acne, alopecia, psoriasis, and fungal infections) due to their anti-inflammatory, antiseptic, and antioxidant properties [1,2]. For instance, traditional usage of pines in treating inflammatory skin conditions, such as abscesses, burns, dermatitis, psoriasis, etc., has been reported in Turkish folk medicine [3]. In the ethnobotany of Bosnia and Herzegovina, original medicinal balms made from the resin of species from the Pinaceae family are prevalent. *P. nigra* J.F. Arnold, *P. sylvestris* L., *P. mugo* Turra, and the endemic species *P. heldreichii* Christ have been traditionally used in healing various skin diseases [4]. Pines originating from the Indian Himalayas have similar traditional applications. The bark has been used to treat skin impairments, such as ulcers and burns [5]. In the regions of the Southern Balkans and the Eastern Mediterranean, pines have been used in treating hemorrhoids, ulcers, and eczema due to their antibacterial and anti-inflammatory properties [6].

Widespread traditional usage of pines for the treatment of inflammatory skin conditions, on the one hand, and the well-known side effects of the prolonged usage of synthetic anti-inflammatory drugs, such as corticosteroids [7], on the other, have recently popularized investigations of the anti-inflammatory potential of these plants [2,8,9,10,11,12,13,14]. There are a few studies that assess the anti-inflammatory effects of pines on the skin. Ait Atmane et al. found that oil, obtained by cold pressing of *P. halepensis* Mill. seeds, has an anti-inflammatory effect, achieved by inhibiting the expression of NF-kB and pro-inflammatory cytokines [15]. Additionally, essential oils from the cones of *P. pinea* L. and *P. halepensis* have demonstrated effectiveness in wound healing in experimental models, as well as anti-inflammatory activity [16]. However, current research on the essential oils (EOs) derived specifically from green cones of *P. halepensis* remains scarce, and to the best of our knowledge, there are limited data regarding their detailed chemical composition and no reports on their anti-inflammatory properties assessed in in vivo experiments, especially for encapsulated EOs. This represents a notable gap in both phytochemical analysis as well as formulation investigation, particularly considering the increasing demand for effective and stable natural products for dermatological and cosmetic applications.

Essential oils (EOs) represent a combination of a number of easily volatile, lipophilic compounds known for their biological activities, which makes them suitable for the treatment of various health impairments [17]. The anti-inflammatory properties of certain types of EOs categorize them as alternative sources of anti-inflammatory drugs [18]. However, it is known that EOs are chemically unstable and that exposure to oxygen, light, and temperature leads to their evaporation and degradation of biologically active components [19]. In line with this, significant attention is devoted to the protection of EOs from external factors and to ensure their stability and effectiveness at the site of action.

The most common approach is encapsulation of EOs to achieve several goals: to provide protection, controlled release, targeted selectivity, and efficiency; to enhance solubility, bioavailability, pharmacokinetics, and pharmaceutical distribution; and to reduce their toxicity and irritability [20]. For this purpose, liposomes seem attractive due to their specific structure making them suitable as carriers of both hydrophilic and lipophilic substances, including EOs, which are usually incorporated into the lipid bilayer [21]. Due to the structural similarity of the liposomal bilayer with cellular membranes, more effective penetration of active substances through the epidermal barrier is enabled [22]. In addition to serving as carriers for active substances, liposomes also exhibit positive effects on the skin and have confirmed cosmetic benefits: they increase skin hydration, create a protective film, help restore compromised skin integrity, and slow down aging. Additionally, liposomal formulations reduce the frequency and severity of skin irritations often caused by the topical application of EOs [23].

In light of the above-mentioned, the present study aimed to explore the ethnopharmacologically inspired potential of this EO per se and after encapsulation into liposomes (LEOs), with the goal of enhancing its biological activity and stability. An in vivo anti-inflammatory effect of an EO and LEO was evaluated using a carrageenan-induced rat paw edema model, while the chemical composition of the EO was thoroughly analyzed to establish potential correlations with its observed biological effects. Additionally, given that the stability of liposomal dispersions is a key determinant of their therapeutic performance, a comprehensive physico-chemical characterization (particle size, polydispersity index, zeta potential, and encapsulation efficiency) of the LEO formulation was conducted. By addressing the dual challenge of bioactivity and delivery, this study provides novel insights into the therapeutic and formulation potential of *P. halepensis* EOs, contributing to the development of safe and effective natural anti-inflammatory agents for potential use in both the pharmaceutical and cosmetic industry.

## 2. Materials and Methods

### 2.1. Plant Material

The green cones of *P. halepensis* Mill. were collected between July and August 2023 from the area of Neum (Bosnia and Herzegovina). Plant identity was verified, and herbarium voucher specimens were deposited at the Institute of Medicinal Plants Research “Dr Josif Pančić” in Belgrade. Before EO isolation and extract preparation, the plant material was stored at −24 °C.

### 2.2. Preparation of EO

The EO of the *P. halepensis* green cones was isolated from the crushed plant material by distillation using steam in a Clevenger apparatus for 2 h, following the protocol outlined in the Pharmacopoeia Jugoslavica editio quarta [24]. The resulting EO was then extracted with diethyl ether and dried using anhydrous sodium sulfate. Following filtration, the solvent was evaporated under a gentle stream of nitrogen at room temperature to prevent any loss of the EO. The EO content is expressed as a percentage in relation to the dry mass of the plant material. The EO was stored at a temperature of 4 °C until analysis.

### 2.3. Chemical Analysis of EO

Quantitative and qualitative analysis of the EO was performed by gas chromatography (GC) and gas chromatography–mass spectrometry (GC-MS).

#### 2.3.1. Gas Chromatography-GC

GC analysis of the EO was performed using the HP-5890 Series II GC apparatus [Hewlett-Packard, Waldbronn (Germany)], which is equipped with a split–splitless injector and an automatic liquid sampler attached to an HP-5 column (25 m × 0.32 mm and a film thickness of 0.52 μm) and fitted to a flame ionization detector (FID). The flow rate of the carrier gas, which was H_2_, was 1 mL/min, the split ratio was 1:30, the injector temperature was set to 50 °C, and the detector temperature was 300 °C. The column temperature was programmed linearly from 40 °C to 260 °C (using a rate of 4 °C/min) and then isothermally at 260 °C for 10 min. The EO was dissolved in a mixture of chloroform and MeOH and injected in the amount of 1 μL. Area percent reports were used for the quantification analysis; the percentage composition of the EO was computed from GC peak areas.

#### 2.3.2. Gas Chromatography–Mass Spectrometry (GC-MS)

The same analytical conditions used for GC-FID were employed for GC-MS analysis, as well. The column used was HP-5MS (30 m × 0.25 mm and a film thickness of 0.25 μm), and the analysis was performed employing the HP G 1800C Series II GCD system apparatus (Hewlett-Packard, Palo Alto, CA, USA). Helium was the carrier gas, while the transfer line was heated at 260 °C. Mass spectra were acquired employing EI mode (70 eV) in the *m*/*z* range of 40–450. The EO was dissolved in a mixture of chloroform and MeOH and injected in the amount of 0.2 μL. The EO constituents were identified by comparing their spectra to the ones obtained from Wiley 275 and NIST/NBS libraries using different search engines. Calibration was performed using a linear *n*-paraffins mixture (C6–C40) as a standard. Values for retention indices were determined via the calibrated Automated Mass Spectral Deconvolution and Identification System Software (AMDIS ver. 2.1, Gaithersburg, MD, USA), compared to those available in the literature, and used as an additional tool to confirm the MS findings.

### 2.4. Protocol for the Production of Liposomal Dispersions and Nomenclature of the Samples for the In Vivo Study

Liposomal dispersions were prepared using commercially available excipient Phosal 40 IP (Lipoid, Ludwigshafen, Germany), which is the so-called do-it-yourself globular encapsulation system containing 40% non-GMO unsaturated phosphatidylcholine dissolved in organic sunflower seed oil, ready to dissolve the EO. A placebo sample of the liposomal dispersion (“blank” liposomes, i.e., sample L) was prepared with Phosal 40 IP and preserved with purified water using Euxyl PE 9010 (INCI: Phenoxyethanol (and) Ethylhexylglycerin), as indicated in Table 1. For preparation of the samples, an LEO (Table 1) and EO concentration of 1% (*w*/*w*) was used. This concentration was chosen according to the literature data [16]. After weighing the ingredients, they were mixed using a rotor-stator homogenizer (IKA Ultra-Turrax^®^ T25 digital, IKA^®^-WerkeGmbH& Co. KG, Staufen, Germany) for 30 min at 8000 rpm. To prepare the final liposome dispersion, the prepared coarse dispersion was passed through a high-pressure homogenizer (EmulsiFlex-C3, Avestin Inc., Ottawa, Canada) five times at a pressure of 500 bar and a temperature of 25 °C.

For an investigation of the influence of the carrier (liposomes) on biological activity, the anti-inflammatory effect assessment also included a tested pine EO per se (Table 1). Bearing in mind that the EOs are not soluble in water, the EO of *P. halepensis* green cones was dissolved in purified water in the same concentration (1% (*w*/*w*)) as in the prepared liposomes (LEOs) with the help of the solubilizer Polysorbate 20 (sample EOP). To check whether the added solubilizer influenced the tested biological activity, it was dissolved in purified water as well (sample P), as seen in Table 1.

After preparation, liposomal dispersions were kept in glass vials at room temperature. Physico-chemical characterization of liposomal dispersions was performed and included EO encapsulation efficiency and measurements of droplet size, zeta potential, polydispersity index, conductivity, and pH value, as well as atomic force microscopy (AFM) and Fourier-transform infrared (FTIR) spectral analysis.

### 2.5. Physico-Chemical Characterization of Liposomal Dispersions

#### 2.5.1. EO Encapsulation Efficiency

The encapsulation efficiency (EE) was determined according to the methods reported in the literature, using protamine as a positively charged protein that builds stabile aggregates with liposomes separable by centrifugation, thus leaving the unencapsulated active substance (essential oil) in the supernatant [25]. Therefore, in order to obtain the supernatant, 2 mL of the liposome sample (LEO) was mixed with the same amount of a protamine solution (10 mg/mL) and vortexed for 60 s. After that, 4 mL of normal saline (Hemofarm, Vršac, Serbia) was added, and the obtained mixture was centrifuged at 3000 rpm for 60 min. To obtain sediment (after liposome destruction), 100 mL of the liposome sample was dissolved in 5 mL of ethanol. To determine the EE of the EO, the absorbance of the free EO dissolved in the supernatant was measured at 260 nm using a UV-VIS spectrophotometer (Thermo Fisher scientific, Waltham, MA, USA). The concentration of the free EO was calculated using a calibration curve with a series of EO solutions [26].

The encapsulation efficiency was calculated as follows:

EE% = (T − S) × 100(1)

where T is the total amount of EO in the supernatant and sediment (after liposome destruction), and S is the amount of EO detected in the supernatant.

#### 2.5.2. Determination of Mean Diameter and Polydispersity Index by Dynamic Light Scattering Method

For drop size analysis of the L and LEO samples, the batch-mode method of dynamic light scattering (DLS) was applied according to the operating procedure NCL-PCC-1 [27] using a Zetasizer Lab Blue Label (Malvern Panalytical, Malvern, UK). The tested liposome samples were diluted in a ratio of 1:500 with 0.01 M of a NaCl solution prepared with dH_2_O immediately before the measurement. Measurements for each sample were performed in three replicates at a temperature of 25 °C.

#### 2.5.3. Determination of Surface Charge of Liposomes

The zeta potential of the L and LEO samples, as an indicator of surface charge, was determined using the Zetasizer Lab Blue Label device (Malvern Panalytical, Malvern, UK) by determining the electrophoretic mobility of liposomes in an electric field. The samples, diluted in a ratio of 1:500 with 0.01 M of a NaCl solution prepared with dH_2_O immediately before the measurement, were transferred into a measuring cell with two electrodes. Measurements were performed in three replicates at 25 °C for each sample.

#### 2.5.4. Determination of pH Values

A digital pH meter (Mettler Toledo, Columbus, OH, USA), calibrated before use with standard buffer solutions (pH 4.0, 7.0, and 9.0), was utilized to determine pH values. Measurements of the pH value of prepared liposome formulations were determined by direct immersion of the appropriate pH meter electrode in the test sample. The examined formulations were L and LEO (Table 1).

#### 2.5.5. Determination of Electrical Conductivity

A conductivity device, Eutech CON 700 (Thermo Fisher Scientific, Shanghai, China), was used to determine electrical conductivity. Measurements of electrical conductivity of the prepared liposome formulations (L and LEO) were determined by direct immersion of a conductometer probe in the test sample. The probe was calibrated with 0.01 M of a KCl solution at room temperature before operation.

#### 2.5.6. Preliminary Physico-Chemical Stability of Liposomes

To check the physico-chemical stability of the prepared liposome samples, measurements of pH value, electrical conductivity, particle size, and zeta potential were evaluated 7 days after preparation and after 1 and 3 months of storage in a glass vial at room temperature.

#### 2.5.7. Fourier-Transform Infrared (FTIR) Spectral Analysis

FTIR spectra were measured directly on the applied samples (L and LEO) using an Agilent Technologies Cary 630 FTIR. The FTIR spectral range mode was from 400 to 4000 cm^−1^, with a resolution of 4 cm^−1^.

#### 2.5.8. Atomic Force Microscopy (AFM)

For AFM analysis, the samples were prepared by diluting them 500 times in 0.01 M of a diluted NaCl solution prepared with deionized water, followed by deposition onto a substrate and drying in a vacuum oven for 1 h (40 °C, 70 mbar). The surface topography of the samples was characterized by an Atomic Force Microscope (NT-MDT Ntegra SPM, Limerick, Ireland) in Semicontact Error Mode. The scanning frequency during the measurements was maintained at 0.5 Hz, while the cantilever oscillation amplitude (i.e., Set Point) during the measurements was 10 V. The images acquired in the magnitude mode (“Mag”) are related to the error signal, providing a higher contrast for sharp details within the scanned area.

### 2.6. In Vivo Experiments

#### 2.6.1. Ethical Statement

This investigation was conducted at the Center for Preclinical and Functional Investigations of the Faculty of Medical Sciences, University of Kragujevac, Serbia. The study protocol was performed in accordance with the regulations of the Faculty’s ethical committee for the welfare of laboratory animals and the principles of the Good Laboratory Practice and European Council Directive (86/609/EEC).

#### 2.6.2. Animals

Adult male Wistar albino rats were obtained from the Military Medical Academy, Belgrade, Serbia, and housed under a controlled temperature of 22 ± 2 °C, with 12 h of automatic illumination daily. Animals consumed commercial rat food (20% protein rat food; Veterinary Institute Subotica, Serbia) and water ad libitum.

#### 2.6.3. Evaluation of Anti-Inflammatory Activity

In vivo assessment of the anti-inflammatory activity of liposome formulations was conducted in a carrageenan-induced rat paw edema model. Acute inflammation was induced by an intraplantar injection of 0.5 mL of 1% carrageenan saline in the left hind paw [28]. The rats (8 weeks old and with a weight of 180–210 g) were divided into 6 groups, depending on the applied topical formulation (6 per group):

L—rats treated with empty liposomes;

LEO—rats treated with liposomes with EO;

EOP—rats treated with essential oil dissolved in purified water with Polysorbate 20;

P—rats treated with a (purified) water solution of Polysorbate 20;

HC—rats treated with a hydrocortisone ointment 1%;

CTRL—untreated rats.

Investigated preparations were applied topically in a quantity of 0.3 g using a spatula and gently rubbed. Sixty minutes after the dermal application of the preparations, the carrageenan was applied. Quantification of the anti-inflammatory effect of the applied preparations was performed by measuring the tissue thickness of the left paw of each rat. The paw tissue thickness was measured at the following time points: immediately before inducing inflammation (time 0), as well as after the first, second, third, and fourth hour following carrageenan injection (times 1, 2, 3, and 4). A digital caliper placed at the midpoint of the measured paw was used to measure the tissue thickness. The percentage of inhibition of inflammation was calculated based on the following formula [28]:

% inhibition = 100 × (1 − At/Ac)(2)

At—average increase in paw thickness observed in the treated group between the two measured time points;

Ac—average increase in paw thickness observed in the untreated (CTRL) group between two measured time points.

### 2.7. Statistical Analysis

IBM SPSS Statistics 23.0 Desktop for Windows (IBM Corporation, Armonik, NY, USA) was used for statistical analysis. All data are expressed as the mean ± standard deviation (SD). The Shapiro–Wilk test was used to assess the distribution of data. For the normal distribution, the data were analyzed by one-way analysis of variance (ANOVA), followed by Tukey’s post hoc test for multiple comparisons. When the distribution was different from normal, the Kruskal–Wallis test was used for comparison between groups. The difference was considered statistically significant when the *p*-value was lower than 0.05.

## 3. Results and Discussion

### 3.1. Chemical Composition of EO

Fourteen components, identified in the *P. halepensis* EO (Table 2), represent 94.55% of the total EO composition. The most abundant compounds detected were *α*-pinene (47.47%), myrcene (14.61%), and (*E*)-caryophyllene (11.70%).

A comparison between the composition of the EO of *P. halapensis* from Corsica and the ones used in our study revealed similarities regarding the main constituents: *α*-pinene (47.5%) was the major component, followed by myrcene (11.0%), (*E*)-caryophyllene (8.3%), and caryophyllene oxide (5.9%) [29]. The major compounds of *P. halepensis* cone EOs from central Italy were α-pinene (53.6%), myrcene (13.7%), and *β*-caryophyllene (6.7%) [30]. Similar findings were reported for the cones of *P. halepensis* from Tunisia, where the main compounds identified were pinene (51.7%) and (*Z*)-caryophyllene (15.05%) [31], while Tumen et al. found *α*-pinene (47,09%), *β*-caryophyllene (11,22%), caryophyllene oxide (7.47%), and *β*-myrcene (6.25%) to be predominant constituents of *P. halepensis* cone EOs [32].

### 3.2. Liposomes Properties

EE represents one of the most important parameters in the liposome encapsulation process [33]. In our study, the EE of the EO in the LEO was 97.35%, confirming the suitability of the developed liposomal formulation. Such results are not surprising, bearing in mind that the excipient used for the production of liposomes (Phosal 40 IP) was recommended by the manufacturer as an encapsulation system for lipophilic actives [34]. Similarly, a study by Ibisevic et al. reported an EE of 84.43% for an oregano EO in a similar liposomal system comprising the same excipient [35].

**Figure 1:**
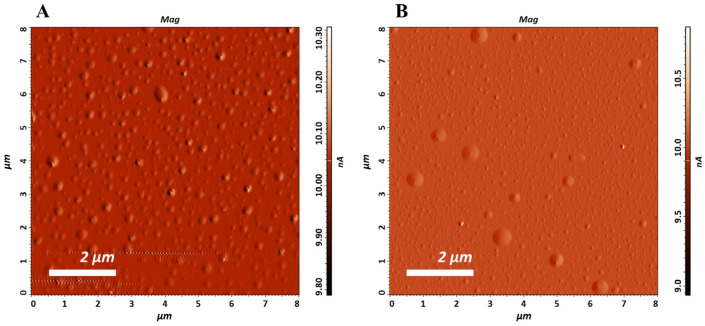
AFM micrographs of the prepared liposomes ((**A**)—empty liposomes and (**B**)—liposomal dispersion encapsulating EO).

The physico-chemical properties of the prepared liposomes (L and LEO) were further investigated by AFM as the method of choice for creating images of the surface of materials at the nanometer level, providing information on size, morphology, and surface texture [36]. AFM analysis confirmed the structure of empty liposomes and liposomes encapsulated in ether oil, providing detailed insights into their morphology and three-dimensional shape. Many spherical or ellipsoidal particles are observed in Figure 1. The size of the spheres was determined by analysis of the image cross-section in the tapping mode. AFM imaging showed that the Ls were nanometric in size with a mean value of 157 ± 32 nm. The mean value of liposomes in sample LEO was 125 ± 31 nm.

The size of LEO liposomes was slightly lower than that of empty liposomes (sample L), indicating that the addition of EO may affect liposome dimensions. Previous studies have stressed the ability of EOs to decrease the sizes of liposomes [37,38,39,40]. It has been hypothesized that this effect was the result of increased cohesion and compression between the nonpolar chains in the membrane liposomes in the presence of EOs [37]. Turina et al. found that monoterpenes in the EOs were positioned at the polar head group region of the bilayer. Such positioning may lead to a reduction in the size of liposomes by causing phosphatidylcholine vesicles to increase the surface curvature of the liposome [41]. Also, Hammoud et al. concluded that essential oils with low solubility can achieve a significant reduction in liposome size. The presence of a hydroxyl group and a low Henry’s constant increases efficiency: less soluble compounds occupy the interior of the lipid bilayer and limit the growth of liposomes [42].

**Figure 2:**
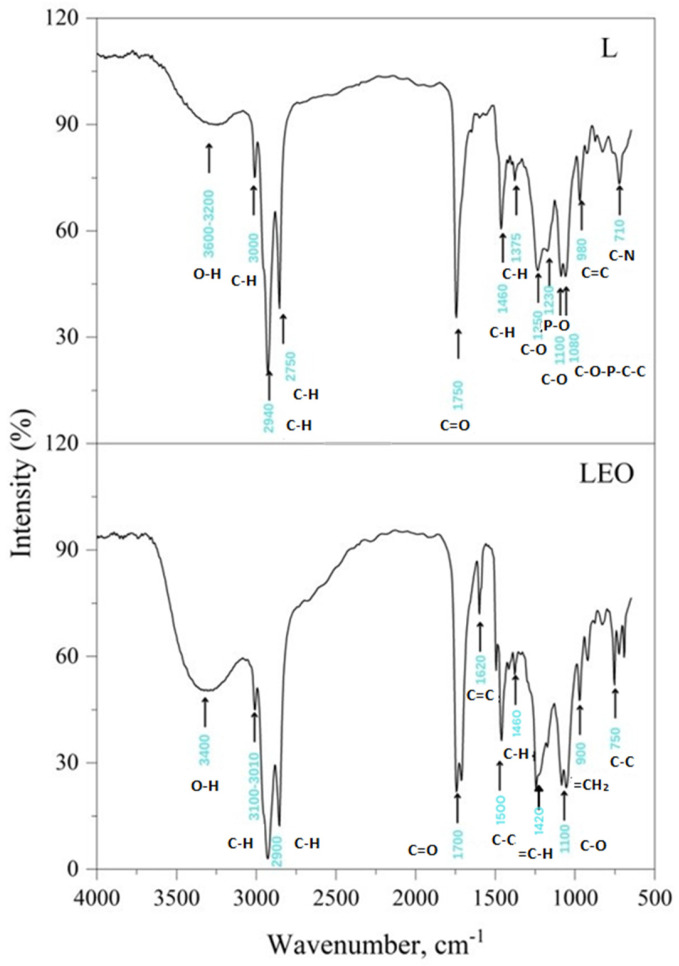
FTIR spectra of the prepared liposomes (L and LEO).

The FTIR spectrum (Figure 2) of free liposomes (L) indicated a broad band between 3600 and 3200 cm^−1^ due to the presence of the hydroxyl group (OH). The minor peak at ~3000 cm^−1^ was assigned to the antisymmetric stretch of terminal CH_3_ groups. Absorption at ~2940 cm^−1^ is specific for the asymmetric stretching vibration in the CH3 groups, while the peak at ~2750 cm^−1^ represented symmetric C–H stretching. The detected peak at 1750 cm^−1^ revealed the stretching vibrations of the ester carbonyl groups (C=O). In the infrared region of lower frequencies, the peak at ~1460 cm^−1^ was associated with scissoring bending vibrations of CH_2_ groups from the lipids, while the absorption at about 1375 cm^−1^ could be related to CH_3_ symmetric bending. At ~1250 and 1100 cm^−1^, specific peaks were detected for the symmetric and asymmetric stretching of the C–O groups. Moreover, typical vibrations of the phosphate groups (in the polar head) were observed for PO^2−^ antisymmetric stretch mode at ~1230 cm^−1^, while the C–O–P–O–C symmetric stretching gave a band at 1080 cm^−1^. The peak at ~980 cm^−1^ corresponded to alkene stretching, which is related to trans disubstitution in the phenyl group, and the peak at ~710 cm^−1^ was assigned to the C–N symmetric stretching of choline.

FTIR spectra proved that there was little difference between the empty liposomes and the encapsulated ones because the majority of functional groups of the EO overlapped with the strong bands of the empty liposomes in the high-frequency region (4000–1500 cm^−1^) and the fingerprint region (1500–1000 cm^−1^). In the FTIR of sample LEO, all expected bands at ~3400 cm^−1^ (broad O–H stretch), ~3100–3010 cm^−1^ (absorption peaks of unsaturation), C–H stretch (~2900 cm^−1^), and C=O and C–O stretch (~1700 cm^−1^ and 1100 cm^−1^, respectively) of terpenoid components were present but covered with the bands belonging to free liposomes. The main components in the EO were α-pinene, myrcene, caryophyllene, and diterpenes. Although the C=CH_2_ in plane deformation vibration of myrcene usually occurs near 1420 cm^−1^, this band was hidden under the CH_2_ and CH_3_ deformation bands near 1460 cm^−1^, but methylene =CH_2_ out-of-plane deformation was seen in the ~900 cm^−1^ region. The intensity of this band in myrcene is high, due to conjugation with the vinyl group, which shows enhanced intensities for the out-of-plane deformation frequencies. The isopropyl or isobutyl groups of caryophyllene should have corresponded to the peaks at ~1466 cm^−1^ and ~1380 cm^−1^, but they were obscured by the bands of free liposomes. Visible at the FTIR spectrum of the LEO were C=C stretches at ~1620 cm^−1^, which might belong both to pinene and caryophyllene (vinyl group, =CH_2_). Most peaks ascribed to diterpenes overlapped with the main bands of free liposomes. Interestingly, the 1700 cm^−1^ band was visible and broader due to the presence of hydrogen bonds between hydroxyls and carboxylic acid groups. In addition, some of the skeletal vibrations of the aromatic ring (C–C stretches) gave a distinct band at ~1500 cm^−1^ and a weak one at ~750 cm^−1^ [33,43].

**Figure 3:**
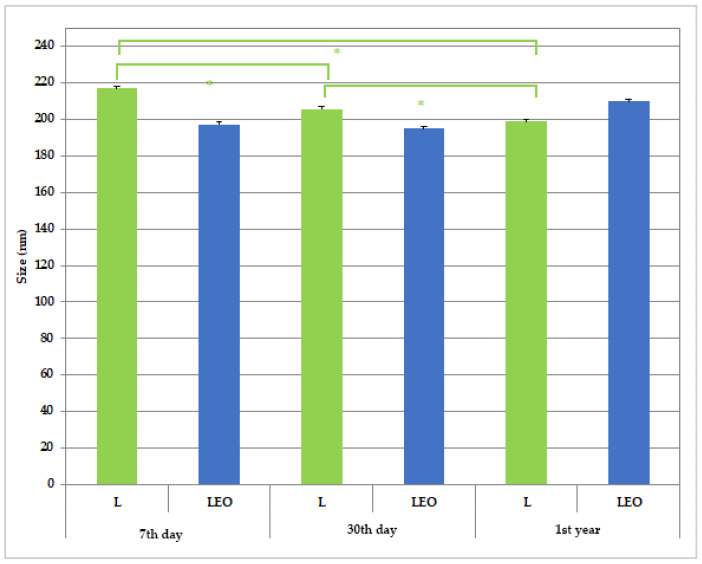
Size of prepared liposomes during 1st year of storage. * A statistically significant difference related to the values of size (between 7th day and 30th day of storage for L; between 30th day and 1st year of storage for L; and between 7th day and 1st year of storage for L).

**Figure 4:**
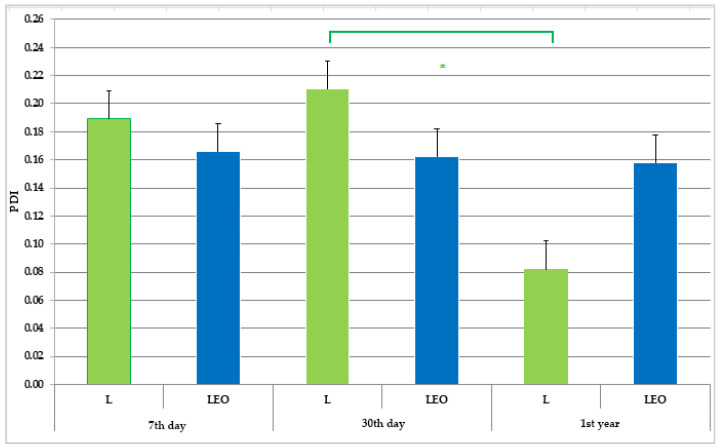
Polydispersity index (PDI) of prepared liposomes during 1st year of storage. * A statistically significant difference related to the values of PDI (between 30th day and 1st year of storage for L).

**Figure 5:**
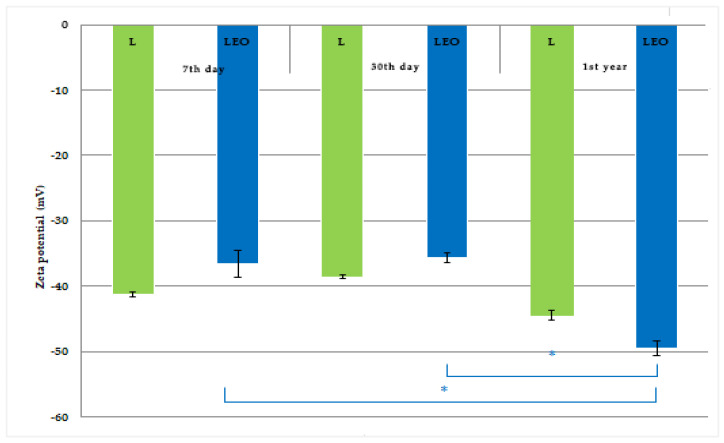
Zeta potential of prepared liposomes during 1st year of storage. * A statistically significant difference related to the values of zeta potential (between 7th day and 1st year of storage for LEO; between 30th day and 1st year of storage for LEO).

The particle size, polydispersity index, and zeta potential of L and LEO are summarized in Figure 3, Figure 4 and Figure 5. The results are presented as the mean value ± SD (*n* = 3), with statistically significant differences set at a level of *p* < 0.01.

A study of the average size and distribution of liposomes is important due to their influence on the stability of the liposomes and also their biological performance, especially when it comes to cutaneous applications [27,39,40].

The droplet size of the prepared liposomes is presented in Figure 3. The particle size values assessed via DLS in the initial measurements were as follows: for empty liposomes, 217.0 ± 1.1 nm, and for encapsulated liposomes, 197.4 ± 3.81 nm. Such findings are in line with previously discussed AFM findings revealing that encapsulation of EO into liposomes leads to a decrease in droplet size.

A particular discrepancy of the results of droplet size obtained via AFM and DLS measurements, due to a larger hydrodynamic diameter of the droplets in the solution (157 ± 32 nm vs. 217.0 ± 1.1 nm for L and 125 ± 31 nm vs. 197.4 ± 3.81 nm for LEO, respectively), may have arisen from the measurement principles of the used methods, due to the larger hydrodynamic diameter of the droplets in the solution when measurements were performed with a zetasizer. Namely, DLS determines the average particle size in the entire solution, with large particles significantly affecting the result. AFM only examines particles adsorbed on the surface, which can deform upon contact with the substrate until the interaction between the vesicle and the surface ends with adhesion after the adsorption process. Additionally, the structure of large liposomal particles can be disrupted during the imaging process itself, which limits the representativeness of the data [44].

The reassessed measurements of particle size revealed a significant decrease in this parameter after 30 days of storage at room temperature, with the same trend observed after 1st year in comparison to the initially assessed value for sample L (Figure 1). The observed decrease may be explained by subsequent particle restructuring, while it has been reported that during storage, repeated liposome collisions and lipid exchange occur, which may change the overall particle size and structure of vehicles. For instance, the presence of short-chain lipids may disrupt the bilayer and reduce the liposome size. Also, a reduction in liposome size during storage has been proposed to be due to hydrolytic degradation of phospholipids, especially in aqueous formulations, leading to smaller vesicles [45,46].

The polydispersity index (PDI) indicates the distribution and uniformity of particle sizes in a system. A low PDI value typically reflects a high level of uniformity among nanoparticles [47]. It is generally accepted that a PDI value of 0.1 to 0.25 indicates a narrow size distribution, whereas a PDI greater than 0.5 suggests a broad distribution of particle size [48]. In all tested samples, the PDI values consistently remained below 0.2, indicating that the liposomes were relatively monodisperse [49]. The mean value of PDI of empty liposomes did not significantly change after 30 days of storage, while it significantly decreased after 1st year in comparison to the result obtained on the 30th day of investigation (sample L). Taking into account the previously discussed reduction in liposome size in sample L, it could be assumed that the observed trend of size decrease was more pronounced between the 30th day and 1st year of the investigation, bearing in mind that there was no significant change in PDI within the first 30 days of storage. Similar tendencies in size and PDI changes during liposome storage were reported by Pinilla et al. [50]. On the other hand, size and PDI measurements of liposomes encapsulating EO (sample LEO) revealed no changes in the stated parameters throughout the same test period (Figure 3 and Figure 4).

Zeta potential is a critical parameter for assessing the physical stability of liposomal formulations. It represents the electrostatic charge on the surface of vesicles, exerting a re-pulsive force that regulates the stability of liposomes, and helps prevent or minimize aggregation and fusion of liposomal particles [51]. The zeta potential of the tested liposomes was –35.61 and –49.43 mV, for the LEO and L samples, respectively, indicating good kinetic stability. Namely, high values of zeta potential (equal to or greater than ± 30 mV) signify optimal electrostatic stability and prevent agglomeration between liposomal particles [52]. Several studies reporting zeta potential values above ± 30 mV demonstrated long-term stability with no aggregation of particles encapsulating EOs [53,54,55,56]. It is well-known that zeta potential can be influenced by numerous factors, including liposome composition, charged lipids, pH, ionic strength of the hydration media, and production parameters. Charged liposomes are created using cationic and anionic phospholipids, which complement neutral lipids and induce electrostatic repulsion between the layers [57]. Negatively charged liposomes generally exhibit greater stability and have a lower leakage rate compared to positively charged liposomes [58,59]. Repeated measurements of zeta potential revealed a significant increase in this parameter at the end of the experiment compared to the initial value and after 30 days of storage for the encapsulated liposome (sample LEO), while the zeta potential of sample L remained the same during the tested period (Figure 5). Interestingly, the EO-encapsulated liposomes showed significantly higher zeta potential values during 1st year of storage, suggesting that the EO increased the negative charge contributing to the enhanced stability, i.e., higher aggregation resistance. This may be explained by the effect of the EO on the structure of the liposome lipid bilayer leading to a redistribution of the electrified groups, thus increasing the charge on the liposome surface [60].

The pH value of the tested samples (Table 3) indicates their applicability in skin products being close to physiological skin pH values, as recommended for pharmaceutical preparations intended for skin application [59]. Chemical stability refers to the potential changes in composition in the product due to the occurrence of various chemical reactions, such as oxidation, hydrolysis, and polymerization. The pH of the liposomal formulation is very important because any change in its value provides information about the chemical instability of the product or contamination that occurred directly or indirectly during the preparation or storage of the product. Changes in pH also indicate a possible chemical reaction between the raw materials used in the product formulation, which affects the effectiveness and safety of the product [61,62]. The measured values of pH in this study show no significant changes throughout 1st year of storage, indicating a satisfactory level of preliminary chemical stability of the produced liposomes.

The electrical conductivity of the tested liposomes was quite high compared to previously reported investigations of liposomes based on similar excipients [63]. The addition of the EO (sample LEO) led to a decrease in conductivity compared to sample L (Table 3). This may be explained by the increase in lipophilic components (EOs) in sample LEO and the consequent reduction in the proportion of water in the formulation. Reassessed measurements of conductivity indicated no notable changes in the stated parameter.

The physical and chemical stability of the prepared liposome samples was assessed based on a reevaluation of the measured physico-chemical parameters (particle size, PDI, zeta potential, pH, and conductivity) after 30 days (short-term stability) and 1st year (long-term stability) of their storage at room temperature. The results of the repeated measurements (Figure 3, Figure 4 and Figure 5, and Table 3) indicate satisfactory short-term and long-term stability of the LEO.

### 3.3. Anti-Inflammatory Activity

The results of in vivo anti-inflammatory activity performed in this study are shown in Table 4. Across all experimental groups, changes in paw inflammation followed a time-dependent pattern. In the first hour after inducing inflammation, none of the tested samples caused a significant reduction in paw inflammation in the rats. Starting from the second hour, the positive control (HC) and LEO demonstrated a significant reduction in inflammation compared to the untreated group (CTRL), whereas the remaining samples exhibited no notable effect (Table 4).

Inflammation represents the initial response of damaged tissue in various inflammatory conditions, characterized by symptoms such as redness, swelling, increased warmth, and pain at the site of injury. The carrageenan-induced paw edema model is frequently employed to assess the anti-inflammatory potential of novel compounds in vivo, while quantification of the inflammatory response typically involves measuring changes in paw swelling following carrageenan injection into the hind paw. This model reveals two distinct phases of inflammation: an early phase, occurring approximately one hour after carrageenan administration, marked by mast cell release of histamine and serotonin leading to heightened vascular permeability, followed by a later phase, characterized by neutrophil infiltration, prostaglandin production, and edema onset [64,65].

Hydrocortisone, a standard topical agent, exhibits potent anti-inflammatory activity by penetrating the skin and suppressing inflammation. This effect is achieved through inhibition of inflammatory cytokine synthesis, including interleukins and tumor necrosis factor-alpha (TNF-alpha), primarily targeting the resolution phase of inflammation. The modulation of gene transcription factors by hydrocortisone results in a decreased inflammatory response [66].

This study represents the first evaluation of the anti-inflammatory effects of pine EO incorporated into a liposomal formulation. Treatment with EO dissolved in purified water with the addition of the solubilizer (sample EOP) led to a high percentage of paw edema inhibition throughout the experiment, reaching a maximum of 44.872% inhibition in the fourth hour. However, the percentage of inhibition was not significant compared to the untreated control but was significantly lower in comparison to the positive control. A similar trend was observed for the empty liposomes (L), as shown in Table 4. On the other hand, LEO demonstrated a statistically significant inhibition of rat paw edema compared to the untreated control during the second phase of carrageenan-induced inflammation (specifically in the third and fourth hours, with 51.83% and 69.23% inhibition, respectively). This phase is marked by neutrophil infiltration at the inflammation site, accompanied by increased production of prostaglandins and other inflammatory mediators [66]. It is important to note that at the end of the experiment, the anti-inflammatory effect of the LEO was comparable to the effect of the positive control, suggesting that the enhancement of efficacy of the tested EO in our study may be attributed to the encapsulation of these compounds into liposomes.

Earlier investigations demonstrated that EOs from *Pinus* species exhibited notable anti-inflammatory properties potentially mediated through the inhibition of inflammatory mediators, like prostaglandins, and modulation of immune cell activity. EOs derived from *Pinus* species contain volatile compounds, like monoterpenes (*α*-pinene and *β*-pinene) or resin acids (abietic and pimaric acid), which can exert anti-inflammatory effects through various mechanisms, including modulation of immune responses and inhibition of inflammatory mediators [67,68]. A study conducted by Süntar and coworkers [16] showed significant anti-inflammatory effects of EOs from cones and needles of different pine tree species (*P. halepensis*, *P. pinea*, *P. sylvestris*, and *P. brutia*) on mice with the acetic acid test and wounded rats. They assumed that α-pinene, as the main constituent of these EOs, was responsible for this effect. The chemical composition of the EO used in this study with α-pinene as the main constituent (Table 2) is in line with the presented literature data.

## 4. Conclusions

In this study, *Pinus halapensis* EO was successfully encapsulated into liposomes, according to FTIR and AFM analyses. The determined EE was high, additionally confirming the suitability of the chosen carriers for the encapsulation of the tested EO. AFM indicated the presence of spherical liposomes of nanoscale, which was confirmed by the average droplet size assessed via a zetasizer, which also suggested relatively monodisperse systems (PDI values below 0.2). The high value of zeta potential (above 30 mV) of the tested liposomes revealed good kinetic stability, while the pH value was in the range suitable for skin application. The repeated measurements of the selected physico-chemical parameters of the tested liposome dispersions showed satisfactory short-term and long-term stability.

Our findings reveal strong anti-inflammatory activity of the LEO, thus advocating for its potential application as a topical anti-inflammatory product. Encapsulation of the EO into liposomes enhanced the anti-inflammatory activity, which was comparable to the effects achieved after treatment with hydrocortisone, used as the positive control.

Overall, the developed liposomal formulation showed good stability and promising anti-inflammatory effects. However, clinical trials are needed to explore the potential use of the formulated liposomal dispersion in the local treatment of inflammatory skin conditions.

## Figures and Tables

**Table 1:** Composition labeling of the prepared samples.

Sample	Sample Label	Phosal 40 IP	EO from *P. halepensis* Green Cones	Polysorbate 20	Euxyl PE 9010	Purified Water	Total
“Blank” liposomes	L	10	-	-	1	89	100
Liposomes with EO from *P. halepensis* green cones	LEO	10	0.5	-	1	88.5	100
Purified water with EO from *P. halepensis* green cones and Polysorbate 20	EOP	-	0.5	2	1	96.5	100
Purified water with Polysorbate 20	P	-	-	2	1	97	100

**Table 2:** Chemical composition of the EOs isolated from green cones of *P. halepensis* used in this study (chief chemical constituents present with a percentage > 0.3).

Chemical Compounds	RI *	CAS	*P. halepensis* EO (%) **
*α*-pinene	932	80–56–8	47.47
camphene	946	79–92–5	0.65
*β*-pinene	974	127–91–3	3.85
myrcene	988	123–35–3	14.61
*δ*-3-carene	1008	13466–78–9	5.10
limonene	1024	138–86–3	5.98
bornyl acetate	1287	76–49–3	0.64
(*E*)-caryophyllene	1417	87–44–5	11.70
*α*-humulene	1452	6753–98–6	2.01
*δ*-cadinene	1522	483–76–1	0.45
caryophyllene oxide	1582	1139–30–6	0.80
manool oxide	1987	596–84–9	0.45
abietatriene	2055	19407–28–4	0.34
dehydroabietal	2274	13601–88–2	0.50
**Total**			94.55
Monoterpene hydrocarbons			77.66
Oxygenated monoterpenes			0.64
Sesquiterpene hydrocarbons			14.16
Oxygenated sesquiterpenes			0.8
Diterpenes			1.29

* retention index; ** area percent reports.

**Table 3:** pH and conductivity of prepared liposomes during three months of storage.

Sample	pH			Conductivity (μs/cm)		
	7th day	30th day	1st year	7th day	30th day	1st year
L	5.00	4.41	4.27	500	519	507
LEO	5.01	4.68	4.35	300	320	350

**Table 4:** Anti-inflammatory effect of liposomal formulation with *Pinus* sp. green cone essential oil in the carrageenan-induced rat paw edema model.

Rat Paw Thickness (mm) (%Inhibition)					
Experimental Groups	0 h	1 h	2 h	3 h	4 h
L	4.40 ± 0.00	5.90 ± 0.16 (16.667%)	6.97 ± 0.73 (25.962%) **	6.50 ± 0.5 (32.258%) **	6.07 ± 0.45 (35.897%) **
LEO	4.40 ± 0.29	5.87 ± 0.05 (18.519%)	5.93 ± 0.42 (55.769%) *	5.89 ± 0.26 (51.828%) *, **	5.20 ± 0.08 (69.231%) *
EOP	4.30 ± 0.08	5.60 ± 0.08 (27.778%)	6.83 ± 0.05 (26.923%) **	6.37 ± 0.09 (33.333%) **	5.73 ± 0.12 (44.872%) **
P	4.00 ± 0.08	5.63 ± 0.05 (9.259%)	7.43 ± 0.12 (0.962%) ** ‡	7.00 ± 0.22 (3.226%) **	6.33 ± 0.12 (10.256%) ** ‡
HC	4.13 ± 0.12	5.33 ± 0.17 (33.333%)	4.73 ± 0.05 (82.692%) *	4.60 ± 0.08 (84.946%) *	4.40 ± 0.08 (89.744%) *
CTRL	4.10 ± 0.08	5.90 ± 0.08	7.57 ± 0.05	7.20 ± 0.08	6.70 ± 0.14

L—rats treated with an empty liposomes; LEO—rats treated with liposomes incorporated with essential oil; EOP—rats treated with water containing essential oil and Polysorbate 20; P—rats treated with a water solution of Polysorbate 20; HC—rats treated with a hydrocortisone ointment 1%; CTRL—untreated rats. Results are presented as the mean value ± SD (*n* = 6). * A statistically significant difference at the level of *p* < 0.05 in relation to the control group; ** a statistically significant difference at the level of *p* < 0.05 in relation to the HC group; ‡ a statistically significant difference at the level of *p* < 0.05 in relation to the LEO group.

## Data Availability

The original contributions presented in this study are included in the article. Further inquiries can be directed at the corresponding author(s).
